# Alternative Protein Sources in the Diet Modulate Microbiota and Functionality in the Distal Intestine of Atlantic Salmon (Salmo salar)

**DOI:** 10.1128/AEM.02615-16

**Published:** 2017-02-15

**Authors:** Karina Gajardo, Alexander Jaramillo-Torres, Trond M. Kortner, Daniel L. Merrifield, John Tinsley, Anne Marie Bakke, Åshild Krogdahl

**Affiliations:** a Department of Basic Sciences and Aquatic Medicine, Faculty of Veterinary Medicine, Norwegian University of Life Sciences (NMBU), Oslo, Norway; b Aquaculture and Fish Nutrition Research Group, School of Biological Sciences, Plymouth University, Plymouth, United Kingdom; c BioMar, Ltd., Grangemouth, United Kingdom; University of Helsinki

**Keywords:** gut microbiota, salmon microbiota

## Abstract

The present study aimed to investigate whether alternative dietary protein sources modulate the microbial communities in the distal intestine (DI) of Atlantic salmon, and whether alterations in microbiota profiles are reflected in modifications in host intestinal function and health status. A 48-day feeding trial was conducted, in which groups of fish received one of five diets: a reference diet in which fishmeal (diet FM) was the only protein source and four experimental diets with commercially relevant compositions containing alternative ingredients as partial replacements of fishmeal, i.e., poultry meal (diet PM), a mix of soybean meal and wheat gluten (diet SBMWG), a mix of soy protein concentrate and poultry meal (diet SPCPM), and guar meal and wheat gluten (diet GMWG). Samples were taken of DI digesta and mucosa for microbial profiling using high-throughput sequencing and from DI whole tissue for immunohistochemistry and expression profiling of marker genes for gut health. Regardless of diet, there were significant differences between the microbial populations in the digesta and the mucosa in the salmon DI. Microbial richness was higher in the digesta than the mucosa. The digesta-associated bacterial communities were more affected by the diet than the mucosa-associated microbiota. Interestingly, both legume-based diets (SBMWG and GMWG) presented high relative abundance of lactic acid bacteria in addition to alteration in the expression of a salmon gene related to cell proliferation (*pcna*). It was, however, not possible to ascertain the cause-effect relationship between changes in bacterial communities and the host's intestinal responses to the diets.

**IMPORTANCE** The intestine of cultivated Atlantic salmon shows symptoms of compromised function, which are most likely caused by imbalances related to the use of new feed ingredients. Intestinal microbiota profiling may become in the future a valuable endpoint measurement in order to assess fish intestinal health status and effects of diet. The present study aimed to gain information about whether alternative dietary protein sources modulate the microbial communities in the Atlantic salmon intestine and whether alterations in microbiota profiles are reflected in alterations in host intestinal function and health status. We demonstrate here that there are substantial differences between the intestinal digesta and mucosa in the presence and abundance of bacteria. The digesta-associated microbiota showed clear dependence on the diet composition, whereas mucosa-associated microbiota appeared to be less affected by diet composition. Most important, the study identified bacterial groups associated with diet-induced gut dysfunction that may be utilized as microbial markers of gut health status in fish.

## INTRODUCTION

The use of alternative plant-based protein sources to partially replace fishmeal in diets for farmed Atlantic salmon (Salmo salar) is currently common practice in commercial diets (1). However, the use of certain feed ingredients of plant origin is restricted due to the presence of antinutrients that challenge function and health of the gut of fish (2). A number of studies describe a range of responses, including inflammation in the gut of carnivorous fish, especially when soybean meal and other legumes are included in the diets (3–10). Some studies have also investigated modulatory effects of different protein sources on the gut microbiota in salmonids (11–16), but general knowledge regarding interactions between diets, gut microbiota, and fish gut function and health is fragmentary and incomplete. There is little doubt regarding the importance of the gut microbiota for the host (reviewed in reference 17). For example, studies in mammals using culture-independent techniques, including high-throughput sequencing (HTS), demonstrated that gut microbial dysbiosis might be closely related to a number of health disorders, such as obesity and inflammatory bowel disease (18, 19). High-resolution microbiota sequencing has also been used to evaluate the role of diet in shaping the gut microbiota in fish (20, 21), including salmonids such as rainbow trout (Oncorhynchus mykiss) (14, 22, 23) and Atlantic salmon (24, 25). However, only a few of the studies conducted with salmon have investigated the mucosa-associated autochthonous and the more transient or digesta-associated allochthonous microbial communities separately. Our recent study of characteristics of the microbiota along the intestine of Atlantic salmon demonstrated important differences between digesta and mucosa (26), implying that dietary modulatory effects may be masked and therefore overlooked if only digesta-associated microbiota is characterized or if a homogenate of digesta and mucosal tissue is evaluated. In fish, investigations of functional aspects of the gut microbiota have so far focused mainly on the modulatory effect of dietary supplements such as pre- and probiotics. Possible links to variation in gut immune functions and growth performance have been suggested (27–31) and, based on experience from studies with mammals, gut microbiota profiling is expected to be a valuable endpoint measurement in order to assess and understand fish gut health status and the effects of diet.

The work presented here was part of a larger study evaluating the effects of practical Atlantic salmon diets with high contents of alternative protein sources, i.e., soybean meal, more highly processed soy protein concentrate, and guar meal, all from legumes belonging to the Fabaceae family, as well as a relevant animal product poultry meal. Results regarding growth performance, nutrient digestibilities, and intestinal histomorphology are reported elsewhere (32). The main aims of the work presented here were to strengthen knowledge on digesta and mucosa-associated microbial composition in the distal intestine using HTS and to evaluate whether high levels of currently used alternative protein sources (i) modulate the microbial communities in the distal intestine and (ii) modulate distal intestine health status and (iii) whether alterations in intestinal microbiota profiles may cause alterations in host intestinal health status and functionality.

## RESULTS

The presentation and following discussion of the results given below focus on differences observed between the reference diet and each of the experimental diets. Differences between the experimental diets are avoided, since their composition was not balanced for direct comparison.

### Characteristics of the high-throughput sequence data.

After sequence quality filtering, trimming, filtering of the operational taxonomic units (OTU) and discarding cyanobacteria reads, 1,191,799 sequences were retained for downstream analyses. The alpha diversity metric Good's coverage estimator was 0.9818 ± 0.0007 (mean ± the standard error of the mean [SEM]), indicating adequate sequencing depth.

### Gut microbiota in distal intestine (DI) digesta.

**FIG 1:**
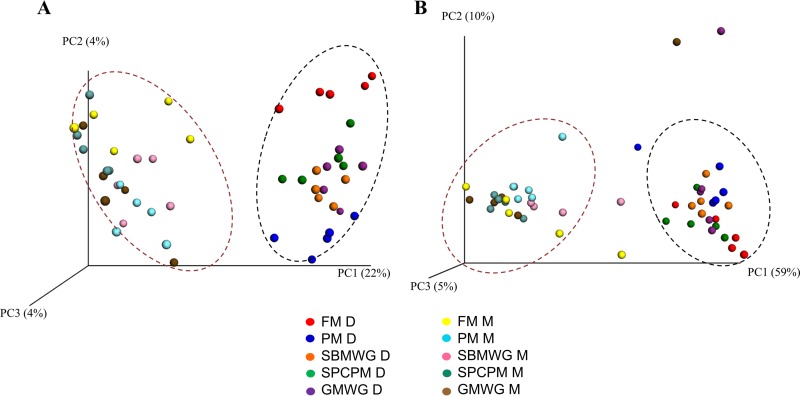
PCoA of unweighted (A) and weighted (B) UniFrac data showing clustering of the digesta and mucosa compartments of the distal intestine of Atlantic salmon fed various diets. Each dot represents one sample. Dotted lines represent the sample origin; the dark red dotted lines indicate clustering mucosa samples, and the black dotted lines indicate clustering digesta samples. FM, fishmeal diet; PM, poultry meal diet; SBMWG, soybean meal with wheat gluten diet; SPCPM, soy protein concentrate with poultry meal diet; GMWG, guar meal with wheat gluten diet; D, digesta; M, mucosa.

Statistical analysis, permutation multivariate analysis of variance (PERMANOVA), of unweighted and weighted UniFrac matrices (Table 1) showed that all diets affected the unweighted UniFrac, indicating that the microbial communities in the digesta of fish fed the experimental diets differed from those in the fishmeal (FM)-fed fish. On the other hand, the weighted UniFrac showed that only fish fed poultry meal (diet PM) and a mix of soybean meal and wheat gluten (diet SBMWG) differed significantly from that of FM-fed fish. The principal coordinate analysis (PCoA) plots of unweighted and weighted UniFrac data (Fig. 1) reflect the statistical analysis by showing clustering of samples by diet, especially in the PCoA plot showing the results of the unweighted UniFrac.

**TABLE 1:** PERMANOVA analysis of weighted and unweighted UniFrac data of DI gut microbiota located in different compartments of Atlantic salmon fed diets with different protein sources

PERMANOVA analysis and diet(s)^*a*^	Unweighted UniFrac		Weighted UniFrac	
	*P*	Pseudo-F	*P*	Pseudo-F
Two way				
Compartments	0.001	13.21	0.001	56.9
Diet	0.007	1.57	0.03	2.19
Interaction	0.213	1.11	0.582	0.83
Pairwise test (digesta)				
FM, PM	0.008		0.046	
FM, SBMWG	0.007		0.01	
FM, SPCPM	0.013		0.254	
FM, GMWG	0.008		0.086	
Pairwise test (mucosa)				
FM, PM	0.013		0.085	
FM, SBMWG	0.025		0.07	
FM, SPCPM	0.275		0.13	
FM, GMWG	0.232		0.396	

a FM, fishmeal diet; PM, poultry meal diet; SBMWG, soybean meal wheat gluten diet; SPCPM, soy protein concentrate poultry meal diet; GMWG, guar meal wheat gluten diet.

**FIG 2:**
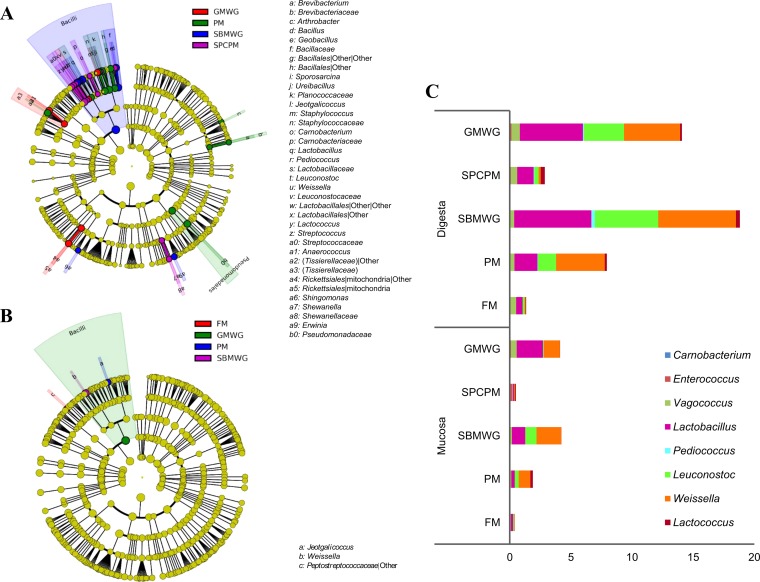
Circular cladogram reporting results from the LEfSe analysis for the identified OTU in the digesta (A) and mucosa (B), and the relative abundance of LAB as percentage of the total OTU found for each diet in digesta and mucosa (C) of the distal intestine of Atlantic salmon fed various diets. In panels A and B, the identified OTU are distributed according to phylogenetic characteristics around the circle. The dots closest to the center represent the OTU on phylum level, whereas the outer circle of dots present the OTU on the genus level. The color of the dots and sectors indicate the compartment in which the respective OTU are most abundant. The color explanation is given in the upper left corner. Yellow color indicates OTU that showed similar abundance in all compartments. The colored sectors give information on class (full name in outermost circle, given only for class showing significant difference between compartments), family, and genera are indicated by letter (explanation given to the right of the panels). Abbreviations are as defined for Fig. 1.

Results from the linear discriminant analysis (LDA) effect size (LEfSe) analysis further support the statistical results, with significant differences in microbial abundances between the fish fed the experimental diets and the FM-fed fish (Fig. 2A). The experimental diets resulted in enrichment of several OTU from different phyla. Compared to all other dietary groups, fish fed SBMWG diet showed a higher abundance of OTU belonging to class Bacilli, genus Bacillus, and the genera Weissella, Leuconostoc, Lactobacillus, Pediococcus, Erwinia, and Sphingomonas. Fish fed PM presented significantly higher abundances of the genera Sporosarcina, Pseudomonadales, Jeotgalicoccus, Arthrobacter, and Brevibacterium. Fish fed a mix of soy protein concentrate and poultry meal (diet SPCPM) presented significantly higher abundances of Streptococcus, Carnobacterium, Lactococcus, Shewanella, Ureibacillus, and Geobacillus, whereas fish fed guar meal and wheat gluten (diet GMWG) presented higher abundances of Anaerococcus and the order Rickettsiales.

**FIG 3:**
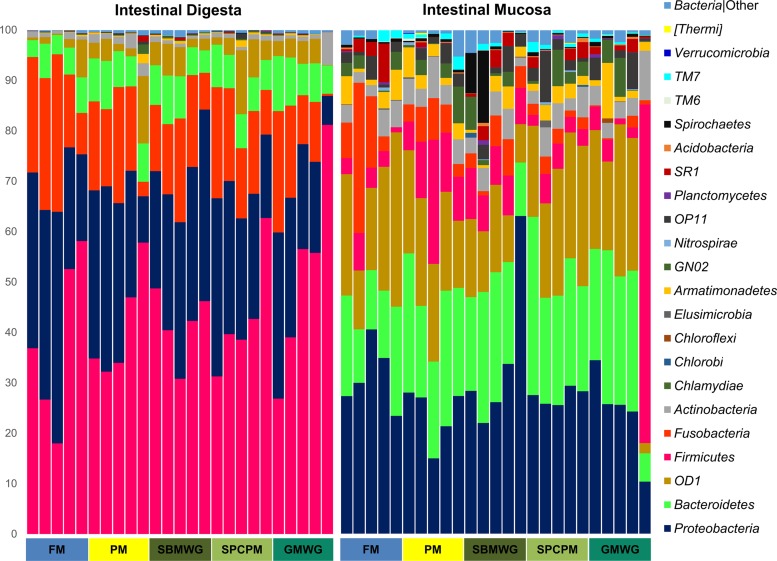
Gut microbiota composition (relative OTU abundance) at the phylum level identified in the distal intestinal digesta and intestinal mucosa samples of Atlantic salmon fed various diets. Abbreviations are as defined for Fig. 1.

Figure 3 shows the relative abundance of OTU at the phylum level. Irrespective of diet, digesta OTU belong mainly to the phyla Firmicutes, Proteobacteria, Fusobacteria, Bacteroidetes, OD1, and Actinobacteria. The digesta of FM-fed fish showed high abundances of Firmicutes (38% ± 17%), Proteobacteria (32% ± 11%), and Fusobacteria (21% ± 9%). In comparison, fish fed the experimental diets presented higher abundances of Firmicutes, from 41% ± 11% in fish fed PM to 52% ± 21% in fish fed GMWG, and lower abundances of Fusobacteria, from 13% ± 9% in fish fed GMWG to 16% ± 5% in fish fed SPCPM and Proteobacteria, from 21% ± 10% in fish fed GMWG to 30% ± 5% in fish fed SBMWG.

**FIG 4:**
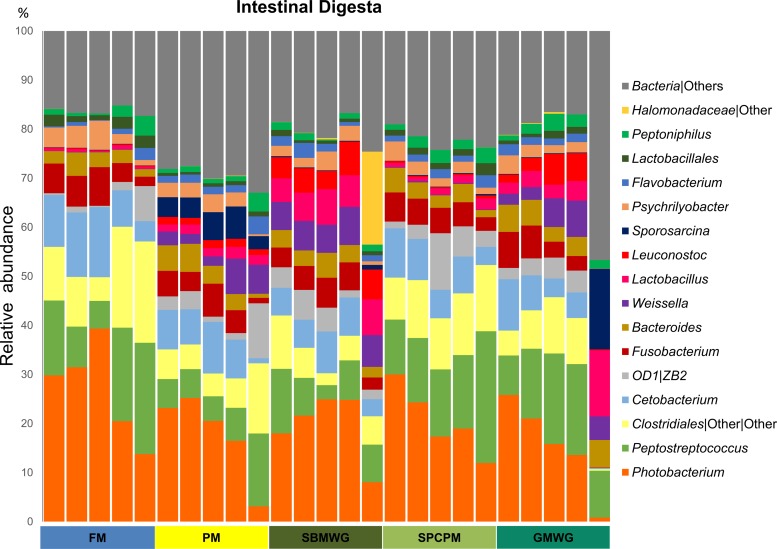
Gut microbiota composition (relative OTU abundance) at the genus level, or the lowest taxonomic level determined by the analysis, of the 17 most abundant genera identified in the distal intestinal digesta samples of Atlantic salmon fed various diets. Abbreviations are as defined for Fig. 1.

Figure 4 shows the relative abundances of the main OTU at the genus taxonomic level in the digesta. FM-fed fish showed a high abundance of Photobacterium (27% ± 10%), Peptostreptococcus (14% ± 7%), Clostridiales (13% ± 7%), and Cetobacterium (10% ± 4%). In comparison, fish fed PM had lower relative abundances of Photobacterium (18% ± 9%), Peptostreptococcus (8% ± 4%), and Clostridiales (7% ± 4%). SBMWG-fed fish also showed lower relative abundance of Photobacterium (19% ± 7%), Peptostreptococcus (8% ± 4%), and Clostridiales (6% ± 3%) but higher abundances of lactic acid bacteria (Fig. 2C) such as Weissella (6% ± 1%), Lactobacillus (6% ± 1%), and Leuconostoc (5% ± 1%) compared to FM-fed fish. Fish fed SPCPM showed a similar relative abundance of Peptostreptococcus (16% ± 6%) and a lower relative abundance of Photobacterium (21% ± 7%) and Clostridiales (11% ± 2%) compared to FM-fed fish. Similar to the SBMWG-fed fish, those fed the GMWG diet showed high abundances of Weissella (5% ± 2%), Leuconostoc (3% ± 3%), and Lactobacillus (5% ± 5%) and lower levels of Photobacterium (15% ± 9%) and Clostridiales (7% ± 4%) compared to FM-fed fish.

### Gut microbiota in DI mucosa.

The results of the PERMANOVA showed significant differences (Table 1) in the unweighted UniFrac between the DI mucosa-associated microbial communities of fish fed the PM and SBMWG diets compared to those fed the FM diet.

LEfSe analysis (Fig. 2B) showed significantly higher abundance of Jeotgalicoccus for fish fed PM compared to the fish fed the other diets. The class Bacilli was significantly more abundant in the GMWG-fed fish than for the other diets. Weissella was more abundant in fish fed SBMWG, and an unidentified OTU from the family Peptostreptococcaceae was less abundant in fish fed the experimental diets than in the fish fed the other diets.

**FIG 5:**
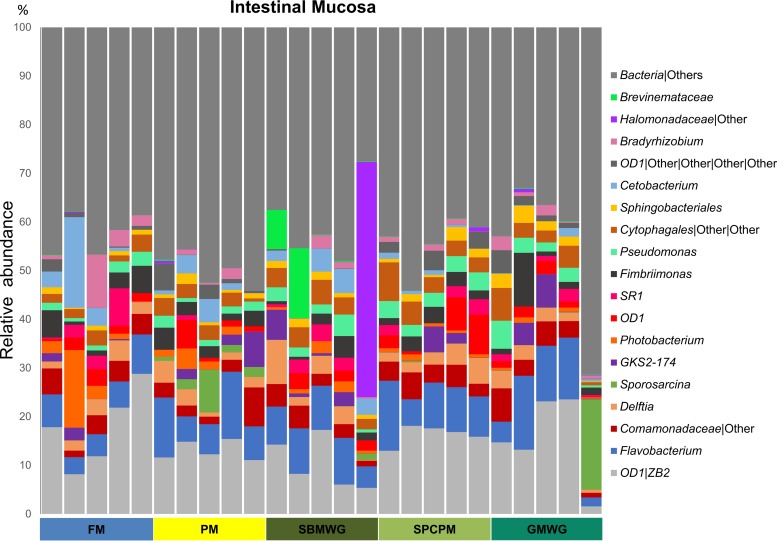
Gut microbiota composition (relative OTU abundance) at the genus level, or the lowest taxonomic level reached by the analysis, of the 19 most abundant genera identified in the distal intestinal mucosa samples of Atlantic salmon fed various diets. Abbreviations are as defined for Fig. 1.

The dominant phyla in the DI mucosa-associated microbiota were Proteobacteria, followed by the phyla Bacteroidetes, OD1, and Firmicutes. Proteobacteria represented 31% ± 7% of the OTU in FM-fed fish and between 24% ± 6% (PM) and 35% ± 16% (SBMWG) for the experimental diets (Fig. 3). The abundances of the other main phyla varied with increases of Bacteroidetes from 15% ± 5% in the FM diet to 20 ± 6 (SBMWG) and 25% ± 6% (SPCPM) in the experimental diets and decreases in Fusobacteria from 11% ± 12% in FM diet to 5 ± 2 (PM) and 1% ± 1% (GMWG) in the experimental diets. Compared to the FM-fed fish, the relative abundance of the phyla OD1 and Firmicutes varied depending on the experimental diet. Higher relative abundance was observed for OD1 in SPCPM (23% ± 4%) and lower relative abundance in GMWG (20% ± 11%) fed fish, PM (19% ± 3%) and SBMWG (12% ± 4%) fed fish compared with FM-fed fish. Firmicutes were more abundant in SBMWG (8% ± 1%), PM (12% ± 7%) and GMWG (16% ± 29%) fed fish compared to FM-fed fish (4% ± 2%). At genus level, the OTU assigned to the mucosal samples (Fig. 5) also showed differences when the experimental diets were compared to the FM diet, but the differences seemed to be smaller than those observed for the digesta samples. The FM-fed fish showed high relative abundance of ZB2 (18% ± 8%; class, no lower taxonomic classification possible), Cetobacterium (5% ± 8%), and Flavobacterium (6% ± 2%). Similarly, fish fed the experimental diets showed a high relative abundance of ZB2 from 10% ± 5% in SBMWG to 16% ± 2% in SPCPM but higher relative abundances of Flavobacterium from 8% ± 2% in SBMWG to 9% ± 3% in SPCPM-fed fish. For a detailed list of OTU, see Data Set S2 in the supplemental material.

### Digesta versus mucosa and core microbiota.

The alpha diversity metrics for richness, observed species, showed significant differences between the two investigated compartments (Table 2), with lower numbers of observed species in samples of mucosa compared to digesta. The observed species parameter of the alpha diversity metric presented higher values for fish fed PM and SBMWG than for FM. On the other hand, the Shannon's diversity index, which takes into account the richness and abundance of the different OTU, did not show significant differences between fish fed the various diets (Table 2).

**TABLE 2:** Alpha diversity results of DI gut microbiota located in different compartments of Atlantic salmon fed diets with different protein sources

Analysis and parameter^*a*^	Richness (observed species) or diversity (Shannon index)^*b*^
Richness (observed species)	
Two-way ANOVA model	
*P* (model)	0.0002
Pooled SEM	13
*P* values from two-way ANOVA	
Segment	<0.0001
Diet	0.004
Interaction	0.283
Mean of significant observations	
Diets	
FM	269^B^
PM	348^A^
SBMWG	329^A^
SPCPM	320^AB^
GMWG	307^AB^
Sections	
Digesta	344^A^
Mucosa	285^B^
Diversity (Shannon index, nonparametric test)	
*P* (model)	<0.0001
Pooled SEM	0.2
Mean for each diet/section studied	
Digesta	
FM	5.4
PM	6.6
SBMWG	6.3
SPMPM	6.0
GMWG	6.0
Mucosa	
FM	6.7
PM	7.0
SBMWG	6.3
SPCPM	6.9
GMWG	6.5

a Diet abbreviations are as defined in Table 1, footnote *a*.

b Mean values with different superscript letters within a column are significantly different (*P* < 0.05).

Moreover, the statistical analysis of the unweighted and weighted UniFrac showed that microbial communities differed significantly between the digesta and mucosa compartments (Table 1). Supporting the previous statistical analysis, the PCoA plots of the unweighted and weighted UniFrac (Fig. 1A and B) presented clear separation between samples of digesta and mucosa origin.

**FIG 6:**
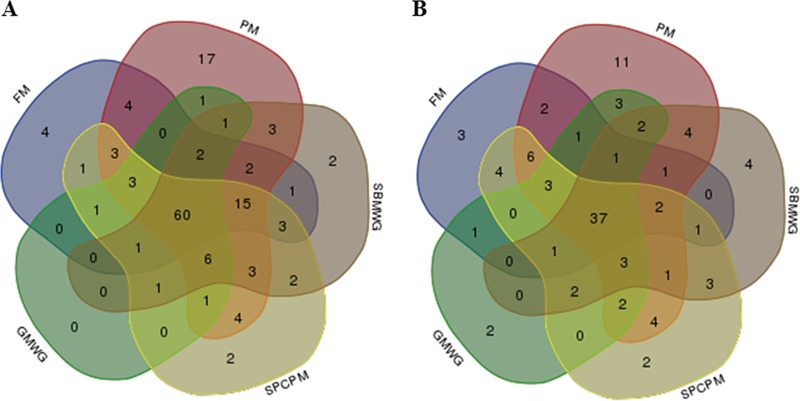
Venn diagrams showing compartmental OTU distributions of the core microbiota identified in the distal intestine of Atlantic salmon fed various diets. (A) OTU distribution in the digesta samples. Sixty OTU were identified as core microbiota (80% of samples) for all diets in the digesta. (B) OTU distribution in the mucosal samples. Thirty-seven OTU were identified as core microbiota (80% of samples) for all diets in the mucosa. Abbreviations are as defined for Fig. 1.

A list of the OTU representing the core microbiota, i.e., those present in 80% of the samples irrespective of diet, is shown in Data Set S3 in the supplemental material. In the digesta, 60 OTU (of a total of 143; Fig. 6A) were observed in all diets groups, showing a dominance of Firmicutes (24 OTU) and Proteobacteria (19 OTU). The core microbiota present in the mucosa was both numerically and proportionally smaller than the core microbiota of digesta, with 37 OTU (of a total of 106) shared by all diets (Fig. 6B). In mucosa, the core was dominated by OTU belonging to Proteobacteria (15 OTU) and Bacteroidetes (9 OTU). The core microbiota for both digesta and mucosa across all samples comprised 19 shared OTU: 6 Bacteroidetes, 5 Proteobacteria, 3 Firmicutes, 3 Fusobacteria, 1 OD1, and 1 Armatimonadetes.

### Tissue gene expression.

The results regarding gene expression of markers for DI tissue function and health are presented in Table 3. Fish fed SBMWG and GMWG showed the greatest modulation in expression levels compared to the FM-fed fish, with a clear increase in *pcna* (proliferating cell nuclear antigen), whereas *frim* (ferritin), and *cat* (catalase) gene expressions were significantly reduced. The SBMWG diet also increased expression of *hsp70* (heat shock protein 70), and a similar trend was seen for fish fed the GMWG diet. In SPCPM-fed fish, the only significant finding was reduced levels of *cat*, whereas no significant changes were observed for fish fed the PM diet. None of the experimental diets caused significant changes in the expression of immune-related genes compared to the FM-fed fish.

**TABLE 3:** Effect of diets with different protein sources on gene expression of distal intestinal tissue of Atlantic salmon

Parameter^*a*^	*P* value, pooled SEM, or mean normalized expression value for various genes^*b*^																
	il-1β*	cd4α*	cd8β*	*gilt*	ifn-γ*	mmp13*	muc2*	*frim*	pcna*	*cat*	hsp70*	myd88*	mhc1*	tcr-γ*	*il-6*†	*il-17a*†	*fabp2a1*†
One-way ANOVA model																	
*P* value	0.17	0.12	0.40	0.09	0.40	0.20	0.26	0.0007	0.0001	<0.0001	0.006	0.02	0.06	0.62	0.32	0.21	0.12
Pooled SEM	0.0006	0.0005	0.0005	1.2	0.0005	0.004	0.97	3	0.03	0.0007	0.4	0.003	0.14	0.0002	0.0002	0.003	0.001
Mean normalized expression values																	
FM	0.0036	0.0030	0.0011	31.0	0.0011	0.029	10.3	51^A^	0.16^B^	0.0078^A^	7.1^B^	0.045^AB^	0.78	0.0023	0.0012	0.004	0.013
PM	0.0030	0.0037	0.0014	30.8	0.0015	0.024	9.3	47^AB^	0.18^B^	0.0063^AB^	7.2^B^	0.046^AB^	0.90	0.0021	0.0009	0.009	0.013
SBMWG	0.0021	0.0040	0.0012	27.6	0.0012	0.037	6.9	35^BC^	0.31^A^	0.0031^C^	8.8^A^	0.052^A^	0.57	0.0018	0.0007	0.011	0.015
SPCPM	0.0016	0.0044	0.0024	31.1	0.0024	0.020	8.2	45^ABC^	0.24^AB^	0.0042^BC^	7.9^AB^	0.047^AB^	0.99	0.0021	0.0005	0.010	0.015
GMWG	0.0022	0.0028	0.0018	27.7	0.0018	0.024	7.3	32^C^	0.32^A^	0.004^BC^	8.4^AB^	0.037^B^	0.56	0.0021	0.0006	0.003	0.015

a Diet abbreviations are as defined in Table 1, footnote *a*.

b As indicated in column 1. *, log-transformed data. IL-1β, interleukin-1β; CD4α, cluster of differentiation 4α; CD8β, cluster of differentiation 8β; GILT, gamma interferon-inducible lysosomal thiol reductase; IFN-γ, interferon γ; MMP13, matrix metallopeptidase 13; MUC2, mucin-2; Frim; ferritin, middle subunit; Pcna, proliferating cell nuclear antigen; CAT, catalase; Hsp70, heat shock protein 70; Myd88, myeloid differentiation factor 88; MHC1, major histocompatibility class 1; TCRγ, T-cell receptor γ. Mean values with different superscript capital letters—A, B, and/or C—within a column are significantly different (*P* <0.05). †, calculated using a nonparametric test.

### PCNA immunohistochemistry.

**FIG 7:**
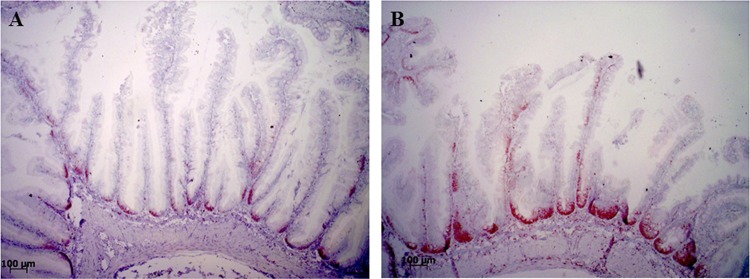
Representative images of the localization and distribution of immunohistochemically labeled PCNA protein in the epithelial cells of the distal intestine of Atlantic salmon. (A) The lowest score in the study, a score of one, with immunopositive cells mostly located in the basal areas of the mucosal folds. (B) The highest score in the study, a score of four, with immunopositive cells reaching up to 75% of the mucosal fold height.

The results of the proliferating cell nuclear antigen (PCNA) staining analysis are shown in Table 4 and Fig. 7. The results of the one-way analysis of variance (ANOVA) (Table 4) showed that, with the exception of the PM diet, all of the experimental diets containing legume products increased the PCNA staining height along the mucosal folds in the DI. The SPCPM-fed fish presented a moderate increase, whereas SBMWG and GMWG diets caused a substantial increase in the number of immunopositive cells along the length of the mucosal folds toward the apex.

**TABLE 4:** Effects of diets with different protein sources on PCNA staining height analysis of distal intestinal tissue of Atlantic salmon

Parameter^*a*^	PCNA measurement^*b*^
One-way ANOVA model	
*P*	<0.0001
Pooled SEM	0.18
Mean PCNA staining score	
FM	1.81^C^
PM	2.00^C^
SBMWG	3.56^A^
SPCPM	2.81^B^
GMWG	3.09^AB^

a Diet abbreviations are as defined in Table 1, footnote *a*.

b Mean values with different superscript letters (A, B, and/or C) within a column are significantly different (*P* < 0.05).

## DISCUSSION

The results from the present study demonstrated that regardless of diet, significant differences between the microbial populations in the digesta and the mucosa were observed in the salmon DI, which is in agreement with similar investigations characterizing the gut microbiota in fish (11, 14, 16, 20, 26, 33, 34). Furthermore, in agreement with Gajardo et al. (26), the alpha diversity metric observed species in the present study showed lower values in mucosa samples than in digesta samples, suggesting that not all bacteria present in the digesta are able to colonize the mucosa of the gut of salmon. Differences observed in the microbial populations between digesta and mucosa have also been reported in terrestrial animals, including humans (35, 36).

In addition, the lower relative abundance of Firmicutes in mucosa compared to the digesta samples was in agreement with observations made in our previous study (26). In line with these results, the OTU reported as core microbiota for all samples belonged mainly to three phyla: Proteobacteria, Firmicutes, and Bacteroidetes. Several of the reported shared OTU, such as Fusobacterium, Microbacterium, Peptostreptococcus, Psychrilyobacter, Pseudomonas, Weissella, Photobacterium, Delftia, and Bradyrhizobium, have been previously reported as members of the gut microbial communities of salmon (16, 26, 34, 37–40). Together, these results suggest that certain bacterial species of some phyla may be more capable of inhabiting the salmon gut despite changes in external environmental factors such as geographical location and diet. It is not yet clear to what extent the host actively plays in selecting or promoting the presence of these core microbes.

Regarding effects of diet composition on gut microbiota, the present results showed the clearest differences between the SBMWG- and FM-fed fish for all studied variables. The differences regarding the lactic acid bacteria (LAB), with the relative abundance being 18 times higher in the digesta of fish fed SBMWG than in FM-fed fish, were possibly the most interesting as these bacteria, with a few minor exceptions, are generally considered to be beneficial for gut health. Lactic acid bacteria comprise genera such as Weissella, Leuconostoc, Lactobacillus, Pediococcus, and Carnobacteria (41). The high abundance observed for Leuconostoc in digesta in the present study is in line with results of our previous work (26), as well as the work of Zarkasi et al. (37). Moreover, Schmidt et al. (25) suggested that LAB from the midintestine (pooled digesta and mucosa) are highly modulated by the diets in a recirculation aquaculture system (RAS) environment. Our results showed that the modulation in LAB observed in the intestine of salmon fed alternative protein sources may be explained by the higher relative abundance of LAB in digesta and not in the mucosa-associated microbiota compared to fish fed fishmeal diets. Previous studies, some employing classical, culture-based methods, have also reported Carnobacterium, another LAB and member of the order Lactobacillales, as highly abundant in the gut of farmed salmon (11, 34, 42–44). In the present study, as well as that of Zarkasi et al. (37) and Gajardo et al. (26), Carnobacterium was only present in minimal abundance. The apparent abundance differences reported between studies of these two related bacteria might be due to differences in fish strains, diet composition, environmental conditions, or sensitivity of the methodologies. A similar trend was observed in the relative abundance of LAB in the mucosa-associated microbiota. LAB accounted for about 4% of the relative abundance in the mucosa of fish fed SBMWG but only 1% in FM-fed fish.

The high abundance of LAB in DI digesta when soybean meal was added to the diet of salmonids has been reported previously (14, 38). The higher level of indigestible fiber and low-molecular-weight oligosaccharides, such as raffinose and stachyose, present in soybean- and other plant-based diets may explain the higher abundance of LAB, known to utilize such substrates for their metabolism and growth. Fish fed GMWG also presented high relative abundances of LAB (14%) in the digesta. The substrate for these LAB may be the water-soluble galactomannans present in guar meal (45). In line with these considerations, fish fed SPCPM, which does not contain low levels of molecular carbohydrates, had low relative abundance of LAB. Whether ingredient processing or other diet components, such as antinutrients, play a role in the way the diet modulates the gut microbiota requires further investigations.

Results from the present study reported elsewhere (32) showed that the DI histomorphology was altered significantly by the SBMWG diet, including alterations typical for soybean meal induced enteritis (SBMIE). The histomorphology appeared normal for fish fed the other experimental diets. The degree of changes in fish fed the SBMWG diet, including the immunological responses reported in the present study, were milder than typically observed when salmon are fed SBM at the level used in the present experiment (3–11, 46). The explanation for differences in responses to soybean meal between experiments may be variations in levels of antinutrients in the batch of soybeans used, in meal processing, diet composition, and processing, in strains of experimental fish, and in feed intake, which may vary with temperature and several other environmental conditions (10, 47, 48). The alterations observed in gene expression in fish fed the SBMWG in the present study, all known to be associated with SBMIE, do indicate a certain impairment of DI health status. Key indicators in this respect are increased cell proliferation, as indicated by the increased PCNA staining and increased *pcna* gene expression, and increased cellular stress, as indicated by induction of *hsp70* gene expression and suppression of *frim* and *cat* gene expression. The direction and magnitude of change of these markers were in accordance with previous reports on SBMIE in salmon (11, 49, 50). Fish fed GMWG and SPCPM showed alterations in some of the functional indicators, however, the effects were small and overall morphology was not altered (32). It is therefore likely that these changes were indicators of normal, physiological adaptations to diet composition.

Since fish fed the SBMWG diet showed high LAB abundance and also showed signs of impaired gut health, the present work might appear to challenge the general understanding that certain bacteria among LAB have positive effects on gut health in fish (reviewed in references 51and 52). On the other hand, the similar increase in LAB observed in the GMWG-fed fish without the presence of signs of inflammation may indicate that LAB increases were a dietary response rather than a cause of or response to inflammation. However, no firm conclusions regarding cause and effect relationships can be made from the current data. To further investigate the significance of LAB for gut health in salmon, studies combining different omics techniques such as metagenomics, transcriptomics, metatranscriptomics, metaproteomics, and metabolomics, as well as the use of gnotobiotic animals, would be expected to supply useful information in this regard. Such techniques have been used successfully in other studies, throwing light on the role of the microbiota and their modulation in disease and health in terrestrial animals, including humans (53–56).

### Conclusions.

The present work confirms our previous work showing clear differences between the digesta and mucosa in the presence and abundance of bacteria. The OTU found in both digesta and mucosa-associated microbiota belonged mainly to the phyla Firmicutes, Proteobacteria, Fusobacteria, Bacteroidetes, and Actinobacteria. In addition, high relative abundance of the phylum OD1 was found in the mucosa-associated microbiota. The diet composition also affected the richness of the gut microbiota, although more so in the digesta-associated than the mucosa-associated microbiota, with plant meals generally increasing abundance and diversity. Fish fed the diet containing soybean meal showed mild distal intestinal enteritis and at the same time a high relative abundance of LAB. Future research should focus on improving our understanding of the functional role of LAB and whether LAB or other bacterial groups may be of importance for the health of the salmon gut.

## MATERIALS AND METHODS

### Experimental fish.

The feeding trial was performed at the RAS research facilities of BioMar in Hirtshals, Denmark, and conducted in accordance with laws regulating experimentation with live animals in Denmark as overseen by the Danish Animal Experiments Inspectorate. For each diet, duplicate, mixed-gender groups of 22 to 23 postsmolt diploid Atlantic salmon mixed sex with an initial mean body weight of 314 ± 2 g (mean ± the SEM) were randomly distributed into 10 0.8-m^3^ fiberglass tanks containing 1,000 liters of seawater. The temperature during the feeding trial was 15°C. Oxygen saturation was above 85% throughout the experiment, and the salinity varied between 32 and 33 g/liter. Continuous lighting of 24 h per day was provided for each tank during the experimental period.

### Diets.

Five diets were formulated, comprising one reference and four with commercially relevant ingredient composition. Table S1 in the supplemental material shows the formulations and chemical compositions of the diets. The reference diet (FM) contained fishmeal as the only protein source (72%). The experimental diets contained one of four different alternative protein sources/mixes replacing a proportion of the fishmeal: 58% poultry meal (PM), soybean meal (30%) mixed with wheat gluten (22%) (SBMWG), soy protein concentrate (30%) mixed with poultry meal (6%) (SPCPM), and guar meal (30%) mixed with wheat gluten (14.5%) (GMWG). Fish oil, rapeseed oil and tapioca were added as lipid and carbohydrate sources to balance the nutrient composition. The diets were supplemented to fulfill the fishes' requirements for lysine, methionine, vitamins, and minerals.

The feeding trial lasted 48 days. Fish were fed continuously by automatic belt feeders during an 18-h feeding period from 1 p.m. to 7 a.m. The uneaten pellets were registered daily to estimate feed intake.

### Sampling.

At termination of the feeding trial, fish were randomly selected for sampling, anesthetized with benzocaine (20 ml/100 liters; Kalmagin, 20%; Centrovet, Santiago, Chile) and then euthanized by cervical dislocation. All sampled fish presented digesta throughout the intestinal tract, considered indicative of intestinal exposure to the diets. For analysis of microbiota, five fish per diet (two and three fish from each of the replicate tanks) were cleaned ventrally with 70% ethanol. The abdominal cavity was then opened at the ventral midline, and the whole intestine was aseptically removed. The DI was chosen as the target region for all measurement, since it is the intestinal region that has shown the greatest alterations when alternative protein sources are included in the diets of salmon. Samples for investigation of the digesta-associated bacteria of the distal intestine (DI) of salmon were collected individually by carefully squeezing the digesta from the intestine into 1.5-ml sterile tubes. Samples for investigation of the mucosa-associated microbiota were collected from DI sections after they were opened and rinsed with sterile phosphate-buffered saline (PBS). Tissue segments of ∼1 cm were sampled from the middle of the DI and subsequently transferred into 1.5-ml sterile tubes. All samples for microbiota analysis were frozen immediately in liquid N_2_ and thereafter stored at −80°C. For RNA extraction, DI tissue samples from nine fish, i.e., from four and five fish from each replicate tank, including the fish sampled for microbiota analysis, were taken and placed in RNAlater (Ambion/Thermo Fisher Scientific, Waltham, MA) at 4°C for 24 h and subsequently stored at −20°C. For immunohistochemical analyses, DI tissue samples from four fish per tank were collected, placed in 4% phosphate-buffered formaldehyde solution for 24 h, and subsequently stored in 70% ethanol until further processing.

### DNA extraction.

DNA was extracted from 200-mg DI digesta samples and 200-mg DI mucosa samples. The extraction was performed using the QIAamp Stool minikit (Qiagen, Crawley, United Kingdom) according to the manufacturer's specification with the following modifications: 1.4 ml of buffer ASL was added to the tubes containing the samples along with 150 mg of glass beads (Merck, Darmstadt, Germany). Then samples were homogenized using the FastPrep-24 instrument (MP Biomedicals, France) at 6.0 m/s two times for 25 s, with a pause of 25 s between the runs. The temperature for the heating incubation was increased from 70 to 90°C, and the incubation time after the addition of proteinase K and buffer AL was increased from 10 to 15 min. DNA concentrations were determined using a NanoDrop 1000 spectrophotometer (Thermo Fisher Scientific, Wilmington, DE).

### PCR amplification and high-throughput sequencing.

To analyze the microbial population of the distal intestinal digesta and mucosa, amplification of the variable regions V1 and V2 of the 16S rRNA was performed. The PCR was conducted using the universal bacterial primers 27F (5′-AGA GTT TGA TCM TGG CTC AG-3′), 338R-I (5′-GCW GCC TCC CGT AGG AGT 3′), and 338R-II (5′-GCW GCC ACC CGT AGG TGT-3′) (57). The reactions were carried out in 50-μl volumes using 1 μl of DNA template, 25 μl of Phusion high-fidelity PCR master mix (Thermo Scientific, CA) and 1 μl of forward and reverse (pooled 338R-I and II) primers (5 μM). The PCR was run as follows: initial denaturation at 98°C for 3 min, followed by 35 cycles of denaturation at 98°C for 15 s, annealing decreasing from 63°C to 53°C in 10 cycles for 30 s, followed in turn by 25 cycles at 53°C for 30 s and an extension at 72°C for 30 s; followed by a final extension at 72°C for 10 min. PCR products were then analyzed in a 1.5% agarose gel and purified using the QIAquick PCR purification kit (Qiagen). High-throughput sequencing of the purified PCR products was carried out using the Ion Torrent Personal Genome Machine system (Life Technologies, California) as described elsewhere (26) using a 318 chip (Life Technologies). Obtained sequences were grouped by sample and filtered within an Ion Torrent Personal Genome Machine software to remove low-quality reads.

### High-throughput sequence data processing.

Bioinformatic analyses of sequence reads were performed after the removal of low-quality scores (Q score < 20 in 80% of the sequences) with FASTX-Toolkit (Hannon Lab). Sequences were concatenated and sorted by sequence similarity into a single fasta file. Sequences were further analyzed using QIIME pipeline (58) as described elsewhere (26), using a length threshold for the multiple alignments of 250 bp. The following software were used in the data processing pipeline: USEARH quality filter pipeline (59), PyNAST (60), RDP classifier (61), phylogenetic tree (62), and UniFrac (63). The Greengenes database (v13.8) was used as a reference database (64). Singletons and OTU with <0.005% abundance were filtered out in order to reduce spurious OTU (65). OTU assigned as cyanobacteria were excluded from the final data set since they were considered to originate from chloroplasts in the ingested content and were therefore not part of the microbiota of the gut (66). QIIME was also used to identify the core microbiota, defined for this study as the OTU present in 80% of the samples of each diet, and to rarefy the OTU tables to calculate alpha diversity metrics (Good's coverage, observed species, and Shannon index) and beta diversity metrics (unweighted and weighted UniFrac). The samples were rarefied to an even sequencing depth of 5,000 per sample since this was the minimum number of reads presented in the samples. The results are generally presented at phylum and genus taxonomic levels or at the lowest taxonomic level assigned for the OTU. The OTU relative abundances for digesta and mucosa samples are given as means ± the standard deviations for each dietary group.

### RNA extraction and qPCR.

RNA purification and quality control, DNase treatment, cDNA synthesis, and quantitative real-time PCR (qPCR) assays were performed as described elsewhere (32). RNA integrity numbers (RIN) were >8 for all samples, with an average RIN of 8.9. A selection of previously proposed marker genes for gut health and metabolism was profiled in DI tissue samples. Primer details are shown in Data Set S1 in the supplemental material. *gapdh*, *rna polymerase II* (*rnapolII*), and *hypoxanthine phosphoribosyltransferase 1* (*hprt1*) genes were evaluated for use as reference genes as described by Kortner et al. (67) and were found to be stably expressed based on their total variation and inter- and intraspecific variance. Thus, the geometric average expression of the *gapdh*, *rnapolII*, and *hprt1* genes was used as the normalization factor. The mean normalized expression of the target genes was calculated from raw *C_q_* values by relative quantification (68).

### Immunohistochemistry.

Immunohistochemistry was performed on fixed DI sections to detect the distribution of the proliferating cell nuclear antigen (PCNA) as described elsewhere (11) with some modifications. Briefly, intestinal tissues were dehydrated and embedded in paraffin. Paraffin-embedded sections (5 μm) were transferred onto glass slides (Super-Frost; Thermo Scientific), dried overnight at room temperature, and incubated for 1 h at 58°C prior to deparaffinization in xylene. The sections where then rehydrated in graded alcohol baths (100, 96, and 70%) and placed in distilled H_2_O. Antigen retrieval was undertaken by heat-treatment in 10 mM citrate buffer at pH 6.0 and 120°C for 15 min. Endogenous peroxidases were blocked by incubating the sections for 40 min at 37°C in 0.05% phenylhydrazine (Sigma-Aldrich, St. Louis, MO).

Nonspecific antibody binding was reduced by incubating the sections for 20 min at room temperature with 5% bovine serum albumin in Tris-buffered saline (BSA/TBS) containing normal horse serum diluted 1:50, followed by overnight incubation at 4°C with the primary antibody (mouse monoclonal anti-PCNA; M0879, Dako Norge, Oslo, Norway) diluted 1:200 in 1% BSA/TBS (11, 69). The sections were then rinsed in PBS and incubated with biotinylated horse anti-mouse secondary antibody diluted 1:200 in 1% BSA/TBS for 20 min at room temperature. According to the manufacturer's instructions, a Vectastain ABC-PO (mouse IgG) kit was used to visualize immunoreactivity. Negative controls were prepared with 1% BSA/TBS instead of the primary antibody. Mayer's hematoxylin was used as a counterstain.

PCNA staining was evaluated blindly in two different distal intestinal sections for each fish using a light microscope. Section evaluation was performed semiquantitatively, scoring the relative height of the immunopositive cells along the mucosal folds between the base and the apex. Score 1 indicated that positive cells were only observed at the base of the mucosal folds, whereas scores 2 to 5 reflect positive cells observed up to ca. 25, 50, 75, and 100% of the total length of mucosal folds, respectively. The average score was calculated from the two sections for each of the eight individual fish sampled per diet.

### Statistical analysis of data.

For sequencing data, the UniFrac distance matrices were analyzed by permutation multivariate analysis of variance (PERMANOVA) with 999 permutations. For this purpose, the dissimilarity matrix for unweighted and weighted UniFrac were exported to the software PRIMER7 with PERMANOVA+ (70). LDA effect size (LEfSe) (71) was used to characterize microbial differences of biological relevance between the diets within the two different compartments. The LEfSe analysis was performed using an alpha value of 0.01 for both the factorial Kruskal-Wallis rank sum test and the pairwise Wilcoxon test and a threshold of 2.0 for the LDA. The approach used was an all-against-all multiclass analysis. The alpha diversity metric Observed species was subjected to a two-way ANOVA analysis with diet and compartment (digesta, mucosa) as class variables. Data were also analyzed by one-way ANOVA with Tukey's multiple-comparison test as post hoc test to aid in the interpretation of the two-way ANOVA results. For gene expression and immunohistochemistry, data were tested for normality and variance homogeneity using the Shapiro-Wilk W test and the Bartlett's test, respectively. The data were then subjected to one-way ANOVA, followed by Tukey's multiple-comparison test using JMP statistical software (v10; SAS Institute, USA). When necessary, the data were transformed to achieve normal distribution (indicated by “*” in Table 3). Since some qPCR data and the Shannon's diversity index did not fulfill the requirement of normal distribution, the analysis was performed using the Wilcoxon/Kruskal-Wallis test, followed by the post hoc Steel-Dwass method to compare the means. The level of significance for all analyses was set at *P* < 0.05.

### Accession number(s).

Microbiota data were exported as individual FastQ files and has been deposited in the Sequence Read Archive of the National Center for Biotechnology Information (SRA, NCBI) under the accession no. PRJNA342252.

## Supplementary Material

Supplemental material
