# Supplementary material for: Alternative Protein Sources in the Diet Modulate Microbiota and Functionality in the Distal Intestine of Atlantic Salmon (Salmo salar)
Source: Appl Environ Microbiol. 2017 Feb 15;83(5):e02615-16. doi: 10.1128/AEM.02615-16 (PMC5311410; doi:10.1128/AEM.02615-16)
Supplement: Supplemental material [file supp_83_5_e02615-16__index.html]

Supplemental material 

# Alternative Protein Sources in the Diet Modulate Microbiota and Functionality in the Distal Intestine of Atlantic Salmon (Salmo salar)

## Supplemental material

- Supplemental file 1 -

  Primer pair sequences (Data Set S1).

  XLSX, 14K
- Supplemental file 2 -

  Relative abundance of all OTU for each sample in digesta and mucosa (Data Set S2).

  XLSX, 125K
- Supplemental file 3 -

  Core microbiota (Data Set S3).

  XLSX, 15K
- Supplemental file 4 -

  Formulations of the experimental diets (Table S1).

  XLSX, 11K
